# Tumor infiltrating lymphocytes (TILs) are a prognosis biomarker in Colombian patients with triple negative breast cancer

**DOI:** 10.1038/s41598-023-48300-4

**Published:** 2023-12-03

**Authors:** Carlos A. Huertas-Caro, Mayra A. Ramírez, Laura Rey-Vargas, Lina María Bejarano-Rivera, Diego Felipe Ballen, Marcela Nuñez, Juan Carlos Mejía, Luz Fernanda Sua-Villegas, Alicia Cock-Rada, Jovanny Zabaleta, Laura Fejerman, María Carolina Sanabria-Salas, Silvia J. Serrano-Gomez

**Affiliations:** 1 Cancer Biology Research Group, National Cancer Institute of Colombia, Bogotá, Colombia; 2 Clinical Oncology Unit. Instituto Nacional de Cancerología and Adjunct Clinical Professor, Pontificia Universidad Javeriana, Bogotá, DC Colombia; 3 Research Support and Follow-Up Group, National Cancer Institute of Colombia, Calle 1 No. 9 -85, Bogotá, DC Colombia; 4 Grupo de Patología, Instituto Nacional de Cancerología, Bogotá, Colombia; 5 Department of Pathology and Laboratory Medicine, Fundación Valle del Lili, and Faculty of Health Sciences, Universidad ICESI, Cali, Colombia; 6 Department of Oncological Breast Surgery and Mastology, Instituto de Cancerología Las Américas, Medellín, Colombia; 7 Department of Interdisciplinary Oncology and Stanley S. Scott Cancer Center, Louisiana State University Health Sciences Center, New Orleans, LA 70112 USA; 8 Department of Public Health Sciences, University of California Davis, Davis, CA USA; 9 UC Davis Comprehensive Cancer Center, University of California Davis, Davis, CA USA

**Keywords:** Breast cancer, CD4-positive T cells, CD8-positive T cells, Cancer microenvironment

## Abstract

Triple negative breast cancer (TNBC) is highly immunogenic and high levels of tumor infiltrating lymphocytes (TILs) have been associated with a better prognosis and higher probability to achieve pathological complete response. Here, we explore the potential role of stromal TILs level and composition as a prognostic and predictive biomarker in TNBC. 195 Tumor biospecimens from patients diagnosed with TNBC were included. Stromal TILs (sTILs), positive CD4/CD8 cells were evaluated. Differences in clinic-pathological characteristics according to immune infiltration were assessed. The predictive and prognostic value of immune infiltration was analyzed by multivariate models. Higher immune infiltration was observed in patients with favorable clinical–pathological features. Survival analysis showed that longer overall survival times were observed in patients with a higher infiltration of sTILs (*p* = 0.00043), CD4 + (*p* = 0.0074) and CD8 + (*p* = 0.008). In the multivariate analysis, low levels of sTILs were found to be associated with a higher mortality hazard (HR: 1.59, 95% CI 1.01–2.48). CD4 and CD8 immune infiltration were associated with higher odds for pathological complete response (OR: 1.20, 95% CI 1.00–1.46, OR: 1.28, 1.02–1.65, respectively). Our results suggest that immune infiltration could be used as a prognostic marker for overall survival in TNBC patients.

## Introduction

Triple-negative breast cancer (TNBC) is characterized by the lack of expression of estrogen receptor (ER), progesterone receptor (PR), and the epidermal growth factor receptor 2 (HER2)^1^. Its prevalence ranges between 15 and 25%, although it has been reported to occur more frequently among young (< 50 years old) Non-Hispanic Black (NHB) and Latina women^2–4^. It is considered the most aggressive breast cancer subtype characterized by earlier relapse and worse survival compared to other breast cancer subtypes^1,5,6^.

Unlike hormone receptor (HR) positive and HER2-positive tumors that highly benefit from endocrine and targeted therapies, TNBC has limited therapeutic options and until recently, cytotoxic chemotherapy was the only systemic therapy approved^7^. The molecular heterogeneity of TNBC has been described by the presence of distinct molecular subtypes, each with different prognosis and possible molecular targets but these data has not changed its clinical management^8–11^.

TNBC tumors are known to be the most immunogenic subtype with relatively high levels of TILs when compared to HR-positive subtypes^12–14^. The presence of high TILs, especially in the tumor proximity, has been considered a surrogate for immune response and has emerged as an important immunological biomarker in breast cancer^8,15^. Recent data have shown TIL density to be both a positive prognostic marker for disease-free and overall survival^16–19^ and a predictive marker for pathologic complete response (pCR) to neoadjuvant chemotherapy^8,20,21^. This beneficial effect might be related to the role of immune cells who are able to identify tumor neoantigens and enhance the adaptive immune response to induce tumoral death^22,23^. Nonetheless, the physiological impact these cells exert on the tumor vary according to their subclassification^17,24^. In this study we sought to evaluate the differences in clinical–pathological variables, clinical outcomes and pCR according to TILs composition and levels to better understand its prognostic and predictive value in Colombian patients.

## Methods

### Patient selection

We conducted a nationwide, multicenter retrospective study series of 195 TNBC patients diagnosed at three health institutions between 2008 and 2016, including the largest reference cancer center in the country, the Colombian National Cancer Institute (NCI) in Bogotá D.C, as well as Fundación Valle de Lili (FVL) in Cali, and Clínica las Américas (CLA) in Medellin. The inclusion criteria were as followed 1) histologically confirmed diagnosis of primary TNBC, 2) availability of formalin-fixed paraffin-embedded (FFPE) tissue blocks from pre-treatment tissue from biopsies and/or surgery (i.e., mastectomies or quadrantectomy for patients that did not receive neoadjuvant chemotherapy) with at least 10% of invasive carcinoma, and 3) availability of clinical–pathological data from the medical records.

TNBC was defined by the lack of ER and PR reactivity (< 1%), and a negative HER2 score (0 + , 1 + , or 2 + with a confirmatory negative result from an in situ hybridization technique), according to the latest American Society of Clinical Oncology/College of American Pathologists (ASCO/CAP) guidelines^25^. Hormone receptors and HER2 expression were reviewed from medical records and re-analyzed by a single pathologist to confirm the diagnosis.

Pathology reports were reviewed to obtain information regarding histopathological diagnosis, lymph node involvement, histological grade, invasion (lymphovascular and/or perineural), and surgical margins. Demographic information, including place of birth, region of origin, as well as clinical data such as age of diagnosis, body mass index (BMI), tumor size, AJCC clinical stage, treatment protocols (neoadjuvant and/or adjuvant treatments), the presence of recurrence and the vital state, were extracted from clinical records at each institution. Pathological complete response (pCR) was evaluated in the cases that had available slides from the surgical procedure following the Chevallier criteria^26^. The study was conducted in accordance with the tenets of the Declaration of Helsinki and was approved by research ethics committee from Colombian National Cancer Institute (approval number: INT­OFI­04956­2018), and each site, and the patients provided written informed consent.

### Immunohistochemistry and TILs assessment

TILs evaluation was performed by a single-blinded pathologist. Stromal TILs evaluation was performed on single full-face hematoxylin and eosin (H&E) slide from a pre-treatment tissue (biopsy or treatment free surgery product) following the International TILs Working Group 2014 guidelines^27^. For each case a single FFPE tissue block with the highest tumoral content was analyzed. sTILs was defined as the percentage of tumoral stromal area that was occupied by mononuclear immunological infiltrate. sTILs was analyzed as a continuous variable and categorized into two groups: high-sTILs (> 10%) and as low-sTILs (≤ 10%). This cut-off point was selected following recommendations from previous studies^21,27,28^ and were defined before statistical analysis.

TILs subpopulations were assessed on 3 µm thick sections from the same pre-treatment FFPE block selected for sTILs evaluation. Monoclonal antibodies for CD4 (clone SP35, Ventana Medical System) and CD8 (clone SP57, Ventana Medical System) were analyzed in a Roche Benchmark XT automated slide preparation system (Roche Ltd., Switzerland). Positive and negative controls were included and 3,3′ diaminobenzidine (DAB) was used as the chromogen. For each sample, three tumoral areas with the highest immune infiltration were selected and digitalized by a microscopy Olympus EP50 camera at an × 400 magnification (× 40 objective). Using the plugin *cell counter* from the Image J program, the number of stromal and intratumoral CD4 + T and CD8 + T cells were counted within the three-chosen fields and averaged to obtain the mean score for each sample. TILs subpopulations were analyzed as continuous variables and categorized into two groups using as a cut-off value the median to define groups of high or low infiltration (supplementary Fig. 1).

### Statistical analysis

Statistical analyses were performed with R-studio (version 4.2.1). Chi-squared and Fisher’s exact test were used to evaluate differences in clinical–pathological characteristics according to sTILs infiltration (high: > 10% vs. low ≤ 10%), along with TILs subpopulations, CD4 + T (high: > 101.33 vs. low: ≤ 101.33, supplementary Fig. 1a) and CD8 + T (high: > 105 vs. low: ≤ 105, supplementary Fig. 1b). Moreover, differences in sTILs infiltration, CD4 + T and CD8 + T subpopulations, by pCR status (pCR vs. no-pCR) were assessed using the Mann–Whitney and T-student test. All statistical tests were two sided and considered significant when *p* ≤ 0.05. Univariate and multivariate binary logistic regression models were used to assess the association of pCR and lymphocytic infiltration. Regarding Odds ratio (OR), we analyzed different increments, as a result was chosen, the increment that better fit to prediction model, per every 10% increase in sTILs, and per every 30 cells increase in CD4 + T and CD8 + T.

Two survival endpoints were evaluated: (1) overall survival (OS) defined as the time interval between diagnosis and death from any cause or last follow-up; (2) Disease-free survival (DFS) defined as the time interval between surgery and the date of recurrence of breast cancer (local, regional, or distant) or last follow up. Differences (OS) and DFS were assessed between the high and low sTILs, CD4 + T, and CD8 + T infiltration groups, using the Kaplan–Meier and log-rank test. A multivariate Cox regression analysis was performed including the following variables: pretreatment nodal status (positive vs. negative) and tumor size (T1 (≤ 2 cm) vs. T2 (> 2 cm)). Hazard ratios (HR) and 95% confidence intervals (95% CI) were calculated for high vs low of sTILs, CD4 + T and CD8 + T.

## Results

### Clinical–pathological characteristics

Clinical–pathological characteristics of patients included are described in Table 1. Most of the patients were diagnosed over the age of 50 (67.7%) and 20.5% were obese at diagnosis. Additionally, most patients were diagnosed at advanced stages (III: 51.1%), presented poorly differentiated tumors (Scarff-Bloom Richardson III: 85.9%), lymph node involvement (53.8%), and had larger tumors (> 2 cm: 80.9%). Concordantly, more than half of the patients underwent neoadjuvant chemotherapy (59.7%), and among this group, 24.7% achieved pCR. The main surgical treatment approach was the modified radical mastectomy (MRM) (53.0%). At the end of the study, 38.9% of the patients had disease recurrence and 53.8% had died.

**Table 1:** Patients’ demographic and clinical–pathological characteristics.

	Entire cohort
	(N = 186)
	N (%)
Institutions	
NCI	124 (66.7)
FVL	49 (26.3)
CLA	13 (7.0)
Clinical characteristic	
BMI	
< 29.9	147 (79.5)
≥ 30	38 (20.5)
No data	1
Age of diagnosis	
≤ 50 years	60 (32.3)
> 50 years	126 (67.7)
Lymph-node involvement	
No	85 (46.2)
Yes	99 (53.8)
No data	2
AJCC Clinical stage	
I-II	91 (48.9)
III	95 (51.1)
Tumor size	
≤ 2 cm	33 (19.1)
> 2 cm	140 (80.9)
No data	13
Neoadjuvant treatment	
No	75 (40.3)
Yes	111 (59.7)
Surgery	
Quadrantectomy	87 (47.0)
MRM	98 (53.0)
No data	1
Pathological characteristics	
Histology diagnosis	
IDC	176 (94.6)
Other	10 (5.4)
Scarff-Bloom Richardson	
I	1 (0.5)
II	25 (13.5)
III	159 (85.9)
No data	1
Histological invasion	
Yes	75 (47.2)
No	84 (52.8)
No data	27
Pathological response	
pCR	21 (24.7)
pNR	17 (20.0)
pPR	47 (55.3)
No received neoadjuvant	75
No data	26
Prognosis-related characteristics	
Recurrence	
No	113 (61.1)
Yes	72 (38.9)
No data	1
Vital status	
Alive	86 (46.2)
Deceased	100 (53.8)

*NCI* Colombian National Cancer Institute*, FVL* Fundación Valle de Lili*, CLA* Clínica las Américas*, BMI* Body mass index, *cm* centimeters, *MRM* Modified radical mastectomy, *IDC* invasive ductal carcinoma, *pCR* pathological complete response, *pNR* pathological no response, *pPR* pathological partial response.

### Clinical–pathological characteristics by lymphocytic infiltration

For this study, we focused on sTILs as they were predominant in our cases and were highly correlated with the number of intratumoral TILs (iTILs) that were present at a lower density (data not shown). High sTILs levels were observed in 44% of the patients. Regarding TILs subpopulations, a high infiltration of CD4 + and CD8 + cells was observed in 50% of the cases for each marker.

We evaluated TNBC patients’ clinical and pathological characteristics according to sTILs infiltration, and by CD4 + T and CD8 + T subpopulations (Table 2). Patients with clinical stage IV were excluded from these analyses. CD4 and CD8 subpopulations were analyzed in a subset of 178 patients due to FFPE tissue depletion. We observed that patients with less than 10% sTILs presented higher clinical stages (III: 63.5% vs. I/II: 36.5%, *p* < 0.001), larger tumors (> 2 cm: 89.5% vs. < 2 cm: 10.5%, *p* = 0.003), and were positive for lymph-node involvement at diagnosis (63.1% vs. negative: 36.9%, *p* = 0.007), compared to patients with more than 10% of sTILs. Similarly, a higher percentage of patients with low sTILs infiltration received neoadjuvant therapy (67.3% vs. no neoadjuvant therapy: 32.7%, *p* = 0.025), of whom 15.8% achieved a pCR, compared to the high sTILs group where 42.9% achieved a pCR (*p* = 0.023). In the same line, more than half of the patients with low sTILs infiltration were already deceased at the end of the study (65.4% vs. alive: 34.6%, *p* = 0.001). Similar results were observed regarding CD4 + T and CD8 + T cells. Patients with lower CD4 and CD8 counts also presented more advanced clinical stages, larger tumors (> 2 cm), and with a higher frequency lymph-node involvement at diagnosis compared to patients with high CD4 + T and CD8 + T counts (Table 2). Furthermore, patients in the low CD4 + T and CD8 + T infiltration groups were also treated more frequently with neoadjuvant therapy and MRM, compared to high CD4 + T and CD8 + T infiltration groups. Interestingly, only patients with high CD4 + T counts seemed to respond significantly better to neoadjuvant treatment (pCR, high: 43.3% vs. low: 16.3%, *p* = 0.006). Only variables that showed statistically significant differences between evaluated groups were included in Table 2.

**Table 2:** Clinical–pathological characteristics according to sTILs, CD4 + T and CD8 + T immune infiltration in TNBC patients.

Characteristics	sTILs			CD4			CD8		
	High (> 10%) (N = 82)	Low (≤ 10%) (N = 104)	*p* value	High (> 101.33) (N = 90)	Low (≤ 101.33) (N = 88)	*p* value	High (> 105) (N = 89)	Low (≤ 105) (N = 89)	*p* value
	N (%)	N (%)		N (%)	N (%)		N (%)	N (%)	
Clinical characteristics									
BMI									
< 29.9	67 (81.7)	80 (77.7)	0.623^a^	68 (76.4)	71 (80.7)	0.610^a^	65 (73.9)	74 (83.1)	0.187^a^
≥ 30	15 (18.3)	23 (22.3)		21 (23.6)	17 (19.3)		23 (26.1)	15 (16.9)	
No data	0	1		1	0		1	0	
Lymph-node involvement									
No	47 (58.0)	38 (36.9)	0.007^a^	53 (59.6)	30 (34.5)	0.001^a^*	52 (59.8)	31 (34.8)	0.002^a^*
Yes	34 (42.0)	65 (63.1)		36 (40.4)	57 (65.5)		35 (40.2)	58 (65.2)	
No data	1	1		1	1		2	0	
AJCC Clinical stage									
I-II	53 (64.6)	38 (36.5)	< 0.001^a^*	63 (70.0)	28 (31.8)	< 0.001^a^*	56 (62.9)	35 (39.3)	0.003^a^*
III	29 (35.4)	66 (63.5)		27 (30.0)	60 (68.2)		33 (37.1)	54 (60.7)	
Tumor size at diagnosis									
≤ 2 cm	23 (29.5)	10 (10.5)	0.003^a^*	25 (31.6)	6 (7.0)	< 0.001^a^*	21 (26.6)	10 (11.6)	0.024^a^*
> 2 cm	55 (70.5)	85 (89.5)		54 (68.4)	80 (93.0)		58 (73.4)	76 (88.4)	
No data	4	9		11	2		10	3	
Neoadjuvant treatment									
No	41 (50.0)	34 (32.7)	0.025^a^*	52 (57.8)	22 (25.0)	< 0.001^a^*	47 (52.8)	27 (30.3)	0.004^a^*
Yes	41 (50.0)	70 (67.3)		38 (42.2)	66 (75.0)		42 (47.2)	62 (69.7)	
Surgery									
Quadrantectomy	48 (60.0)	39 (37.9)	0.005^a^*	59 (67.8)	28 (31.8)	< 0.001^a^*	55 (64.0)	32 (36.0)	< 0.001^a^*
MRM	32 (40.0)	64 (62.1)		28 (32.2)	60 (68.2)		31 (36.0)	57 (64.0)	
No data	2	1		3	0		3	0	
Pathological characteristics									
Pathological response									
pCR	12 (42.9)	9 (15.8)	0.023^b^*	13 (43.3)	8 (16.3)	0.006^a^*	13 (38.2)	8 (17.8)	0.106^a^
pNR	3 (10.7)	14 (24.6)		7 (23.3)	7 (14.3)		6 (17.6)	8 (17.8)	
pPR	13 (46.4)	34 (59.6)		10 (33.3)	34 (69.4)		15 (44.1)	29 (64.4)	
Prognosis-related characteristics									
Vital state									
Alive	50 (61.0)	36 (34.6)	0.001^a^*	55 (61.1)	29 (33.0)	< 0.001^a^*	52 (58.4)	32 (36.0)	0.004^a^*
Deceased	32 (39.0)	68 (65.4)		35 (38.9)	59 (67.0)		37 (41.6)	57 (64.0)	

*TNBC* triple negative breast cancer, *sTILs* stromal tumoral infiltrating lymphocytes, *PD-L1* Programmed Death-ligand 1, *BMI* body mass index, *cm* centimeters, *cCR* clinical complete response, *nCR* nonclinical response, *MRM* Modified radical mastectomy, *pCR* pathological complete response, *pNR* pathological no response, *pPR* pathological partial response.

*p value is significant.

^a^Chi-square test.

^b^Fisher’s exact test.

### sTILs immune infiltration and pathological complete response

**Figure 1:**
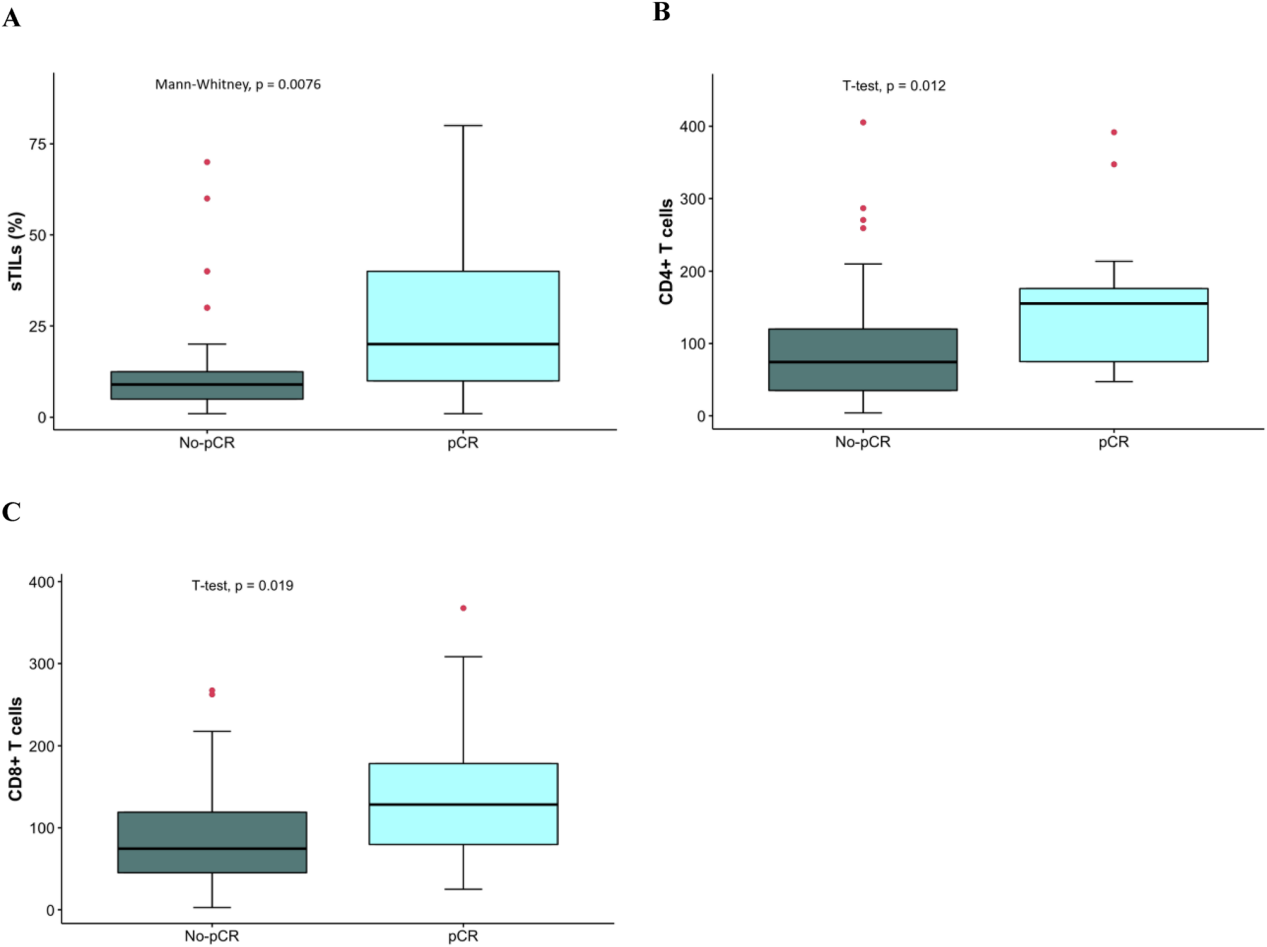
sTILs, CD4 + T and CD8 + T immune infiltration counts according to pCR. Boxplots represent cell counts of (**A**) sTILs, (**B**) CD4 + T and (**C**) CD8 + T according to pathological response (No-pCR or pCR). *sTILs:* stromal tumoral infiltrating lymphocytes.

We compared sTILs, CD4 + T and CD8 + T infiltration levels measured as a continuous variable according to neoadjuvant treatment response, using pCR as the defining variable (Fig. 1). Information regarding neoadjuvant chemotherapy administered to these patients are included in supplementary Table 1. We consistently observed that patients that successfully achieved pCR presented higher sTILs levels (*p* = 0.0076, Fig. 1a), as well as CD4 + T (*p* = 0.012, Fig. 1b) and CD8 + T (*p* = 0.019, Fig. 1c) infiltration, compared to patients that did not achieve pCR.

Moreover, univariate models showed a statistically significant association between pCR and sTILs (OR = 1.48, 95% CI 1.14–2.01, *p* = 0.005) and CD4 and CD8 immune infiltration (CD4 + T: OR = 1.26, 95% CI 1.06–1.53, *p* = 0.012; CD8 + T: OR = 1.33, 95% CI 1.08–1.69, *p* = 0.0094). Consistently, after adjusting the model for lymph-node involvement and tumor size, for sTILs and CD4, the association previously observed was attenuated (sTILs: OR = 1.31, 95% CI 0.984–1.806, *p* = 0.0762; CD4: OR = 1.201, 95% CI 1.003–1.461, *p* = 0.0568) but remained statistically significant for CD8 immune infiltration (OR = 1.277, 95% CI 1.017–1.647, *p* = 0.0433) (Table 3).

**Table 3:** Association of sTILs, CD4 and CD8 immune infiltration and pCR in neoadjuvant-treated TNBC patients.

	Univariate analysis			Multivariate analysis		
	OR	95% CI	*p* value	OR	95% CI	*p* value
sTILs (per 10%)	1.48	1.14–2.01	0.0053	1.310	0.984–1.806	0.0762
CD4 (per 30 cells)	1.26	1.06–1.53	0.012	1.201	1.003–1.461	0.0508
CD8 (per 30 cells)	1.33	1.08–1.69	0.0094	1.277	1.017–1.647	0.0433

*TNBC* triple negative breast cancer, *sTILs* stromal tumoral infiltrating lymphocytes, *pCR* pathological complete response.

### Prognostic value of immune infiltration in TNBC

**Figure 2:**
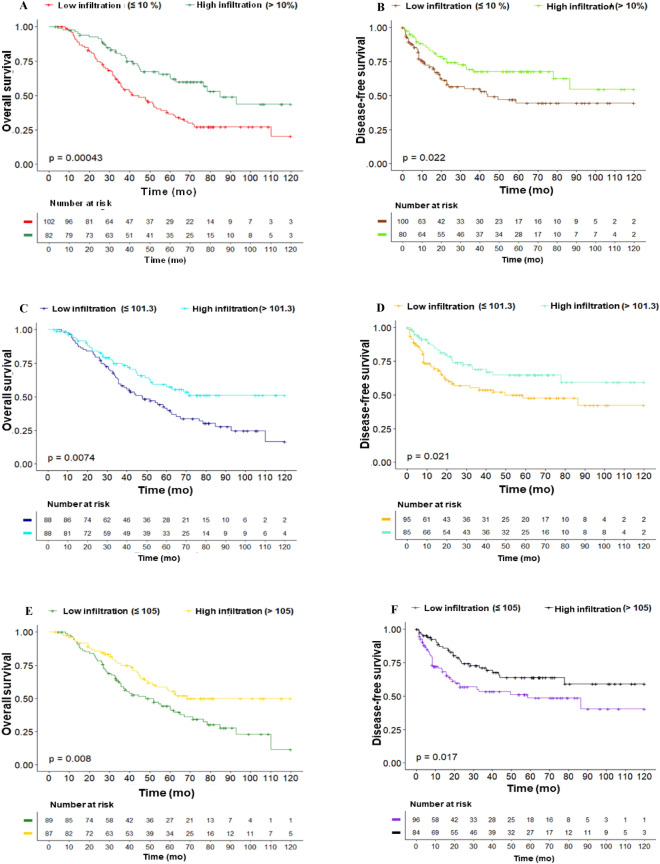
Survival analysis in TNBC patients according to immune infiltration levels. (**A, C, E**) Overall survival and (**B, D, F**) disease-free survival was analyzed according to total (**A–B**) sTILs, (**C–D**) CD4 + T and (**E–F**) CD8 + T immune infiltration. *sTILs* stromal tumoral infiltrating lymphocytes, *mo* months.

Differences in OS and DFS between sTILs, CD4 + T and CD8 + T infiltration groups were analyzed. We observed that patients with low immune infiltration present significantly lower OS and DFS median times compared to TNBC patients with high immune infiltration (Fig. 2).

When sTILs levels was analyzed as a categorical variable, we observed that having less than 10% of sTILs, or lower CD4 + T (< 101.3) and CD8 + T (< 105) cell counts was significantly associated with a higher risk of mortality (HR = 2.08, 95% CI 1.35–3.20, *p* < 0.001; HR = 1.85, 95% CI 1.19–2.89, *p* = 0.007; HR = 1.79, 95% CI 1.16–2.77, *p* = 0.008, respectively) and recurrence (HR = 1.78, 95% CI 1.08–2.92, *p* = 0.023; HR = 1.73, 95% CI 1.05–2.87, *p* = 0.033; HR = 1.77, 95% CI 1.07–2.92, *p* = 0.027, respectively). In a model adjusted by tumor size and lymph-node involvement, we found that having a low sTILs infiltration is an independent prognostic factor for mortality (HR = 1.59, 95% CI 1.01–2.48, *p* = 0.043) (Table 4). Similar results were observed for OS when the model was adjusted by clinical stage (Supplementary Table 2), but there was no association when the model was stratified by clinical (Supplementary Table 3).

**Table 4:** Univariate and multivariate Cox proportional hazards models for mortality and recurrence by sTILs, CD4 + T and CD8 + T infiltration.

		Overall survival		Disease-free survival	
		HR (CI 95%)	*p* value	HR (CI 95%)	*p* value
Univariate model					
sTILs	High	Ref		Ref	
	Low	2.08 (1.35–3.20)	< 0.001	1.78 (1.08–2.92)	0.023
CD4	High	Ref		Ref	
	Low	1.85 (1.19–2.89)	0.007	1.73 (1.05–2.87)	0.033
CD8	High	Ref		Ref	
	Low	1.79 (1.16–2.77)	0.008	1.77 (1.07–2.92)	0.027
Multivariate model					
sTILs	High	Ref		Ref	
	Low	1.59 (1.01–2.48)	0.043	1.37 (0.82–2.27)	0.2
CD4	High	Ref		Ref	
	Low	1.23 (0.77–1.87)	0.4	1.26 (0.72–2.18)	0.4
CD8	High	Ref		Ref	
	Low	1.40 (0.89–2.20)	0.14	1.41 (0.84–2.36)	0.2

The multivariate Cox model included tumor size and lymph-node involvement. *HR:* Hazard Ratio, *CI:* Confidence interval, *sTILs:* stromal tumoral infiltrating lymphocytes.

## Discussion

There is growing interest in analyzing TILs and immune subpopulations in the clinical practice to explore their potential as prognostic and predictive biomarker in a highly aggressive subtype such as TNBC. As has been reported before, tumors with high immune infiltration often present better clinical outcomes and favorable clinical–pathological features^20,23,29,30^. In this study, we analyzed a Colombian cohort of patients with TNBC with a long-term follow-up to explore the immune infiltration and its association with patient outcome and pCR achievement and found sTILs as a robust and independent prognostic marker for mortality and an association with pCR in TNBCs. We believe this is the first study performed in Colombian women to report this finding. This is important as the prevalence of TNBC in our population is higher than the prevalence reported in European-American women^31–33^, and we highlight the need to better understand TNBC in our patients to improve its prognosis and to better identify patients that could benefit from immune-based treatments.

In accordance with previous studies^17,21,34,35^, ours showed an association between clinical–pathological features and the prognostic value of immune infiltration. We observed that patients with high levels of sTILs, CD4 + T, and CD8 + T cells are more frequently diagnosed at earlier clinical stages (I/II), with smaller tumor sizes and no lymph node involvement. Additionally high sTILs were associated with longer OS times and this association remained significant with the inclusion of known clinical variables associated with the prognosis. In that sense, we found that sTILs can be an independent prognostic factor for OS, whereas for CD4 + T and CD8 + T subpopulations, although they were found associated with prognosis in the univariate analyses, the statistical significance was attenuated in the multivariate model.

Immune subpopulations, CD4 + T and CD8 + T, represent only a general fraction of the total population of immune cells that integrate TNBC microenvironment^17^. In that sense, CD4 + T and CD8 + T cell counts are not fully informative and sufficient to establish the effect of immune infiltrate on the disease prognosis, although they might serve as an approximation. A more accurate characterization of the tumor immune infiltration profile should include broader information about other lymphocyte subpopulations with relevant immunological roles^36^.

Even though our results are consistent with the associations reported in other studies^21,35^, the findings around the prognostic value of TILs between studies are still controversial. It is important to take into consideration the stage at diagnosis and lymph node status at diagnosis when analyzing results from different studies as this could impact the association with the prognosis. For example, a study conducted on 133 TNBC patients at earlier clinical stages and small tumor burdens did not find TILs associated with survival outcomes^37^. Presumably, patients at earlier clinical stages and with well-differentiated tumors present lower amounts of tumor antigens and, as result lower immune infiltrate^37,38^. In our study 46.9% of the patients were at stage I/II and 49% at stage III. Moreover 86.6% of the tumors were poorly differentiated.

Higher immune infiltration before neoadjuvant chemotherapy has been significantly associated with higher pCR rates^20,39–41^. In our study population, 23.3% of the patients achieved pCR. This percentage is concordant with the rates reported in other studies where 16.6–48% of TNBC patients achieved pCR. The variability in the percentages could be related with differences in chemotherapy schemes between studies^42–44^. Regarding groups of high or low immune infiltration, we observed that patients with high immune infiltrate received less neoadjuvant chemotherapy and were mostly treated with conservative surgeries but on the other hand a higher percentage of patients with high levels of sTILs, CD4 + T, and CD8 + T achieved pCR. The degree of the sTILs antitumor immune response against cancer cells acts synergistically with natural immunity induced by chemotherapy to restore the cytotoxic response^19,45^ Moreover, chemotherapy can promote an antitumor immune response through the induction of danger associated molecular patterns (DAMP) signals during cell death, in addition to other molecules like the calreticulin (CALR) and the high mobility group release box 1 (HMGB1), which can increase levels of TILs in the TME as in the residual disease after treatment^30,46,47^. sTILs were not clearly shown to have an association with pCR, whereas CD4 + and specially CD8 + cells were found to be associated with this outcome. A possible explanation for that is that different immune populations are included when sTILs are evaluated in H&E slides. For example, Tumor associated macrophages (TAMs) within the TME contribute to evasion and suppression of the immune response and likewise this has been associated with resistance to chemotherapy^48,49^. It should be noted that none of the patients in this study receive immunotherapy. Further investigations are needed to keep exploring the interactions between specific immune cell populations, the tumor phenotype and treatment regimen including immunotherapy, in order to have a better understanding of their role in TNBC.

There is lack of an established standard methodology for immune infiltration assessment. Studies in the field have used different approaches for TILs evaluation to test its association with breast cancer prognosis, managing this variable either as categorical or continuous, and with undefined cut-off values^27^ contributing to the highly heterogenous reports around TILs in breast cancer. In 2014, the International TILs Working Group^27^ published a series of recommendations for TILs assessment, where they came to the consensus that TILs evaluation may provide more accurate information when scored as a continuous variable, given that it would allow a more standard categorization around different thresholds. In the present study, we analyzed TILs both ways, as continuous and categorical variable. Either way, consistent associations were observed, where better clinical–pathological features and longer OS and DFS were found for patients with higher immune infiltration.

The study has some limitations, including the heterogeneity of the specimens and the difference in sample sizes between the three health institutions involved. FFPE blocks were taken from each institution’s pathology archive, therefore, it is possible that sample quality and their general management might have varied considerably from center to center, affecting downstream analyses like the IHC. On the same line, given that we worked with biopsies which are tissue-limited specimens, it was not possible to test additional IHC immune biomarkers to assess a broader spectrum of TILs subpopulations. New methodologies based on tissue microarrays, flow cytometry, and the use of gene expression data are being developed to quantify different immune cells subpopulations^50^. These approaches can be implemented in future studies to improve immune infiltrate assessment and gain a better understanding of TILs effect on TNBC.

## Conclusions

This is the first study in Colombian women to assess immune infiltration as prognostic and potential predictive biomarker in breast cancer patients with TNBC. The results obtained in this study suggest that patients with TNBC, high infiltration of sTILs, and CD4 + T and CD8 + T immune populations, present clinical–pathological characteristics of favorable prognosis. In addition, high levels of immune infiltration were found as an independent factor for overall survival, and a potential biomarker for pCR. However, it is still necessary to continue exploring the relationship between infiltrate immune and prognosis in a higher sample size also including patients from different Colombian regions. Moreover, it is important to evaluate more specific immune marker by different methodologies.

We want to highlight the interdisciplinary work conducted by pathologists, oncologists, molecular biologist, and other scientific professionals, which have enriched this work, contributing this way to the progress of science in Colombia.

### Supplementary Information

Supplementary Information.

## Data Availability

The datasets generated during and/or analyzed during the current study are available from the corresponding author on reasonable request.
