# Supplementary material for: Tumor infiltrating lymphocytes (TILs) are a prognosis biomarker in Colombian patients with triple negative breast cancer
Source: Sci Rep. 2023 Dec 3;13:21324. doi: 10.1038/s41598-023-48300-4 (PMC10694133; doi:10.1038/s41598-023-48300-4)
Supplement: Supplementary file 1 — Supplementary Information. [file 41598_2023_48300_MOESM1_ESM.pdf]

SUPPLEMENTARY TABLE 1

Table supplementary 1. Neoadjuvant treatment in TNBC patients included in the study.

	**N = 195**
	**N (%)**
**Platinum-based neoadjuvant treatment**	
Yes	31 (26.7)
No	85 (73.3)
Did not receive neoadjuvant	78
No data	1
**Anthracycline and Taxanes based Neoadjuvant treatment**	
AC	22 (19.0)
AC+Taxanes	78 (67.2)
Taxanes	16 (13.8)
Did not receive neoadjuvant	78
No data	1

SUPPLEMENTARY TABLE 2

Table supplementary 2. Cox proportional hazards models for mortality adjusted by clinical stage.

			**Overall survival**	
			**HR (CI 95%)**	***P* value**
**Multivariate model**	**sTILs**	High	Ref.	
		Low	1.56 (1.005 – 2.418)	0.047
	**CD4**	High	Ref	
		Low	1.234 (0.79 - 1.928)	0.36
	**CD8**	High	Ref	
		Low	1.491 (0.977 - 2.276)	0.064

SUPPLEMENTARY TABLE 3

Table supplementary 3. Cox proportional hazards models for mortality stratified by clinical stage.

			**Stage I/II**		**Stage III**	
			**Overall survival**		**Overall survival**	
			**HR (CI 95%)**	***P* value**	**HR (CI 95%)**	***P* value**
**Univariate model**	**sTILs**	High	Ref.		Ref.	
		Low	2.072 (0.995 – 4.314)	0.05	1.34 (0.7907 – 2.271)	0.27
	**CD4**	High	Ref.		Ref.	
		Low	1.463 (0.702 – 3.05)	0.3	1.144 (0.663 – 1.974)	0.62
	**CD8**	High	Ref.		Ref.	
		Low	1.375 (0.6597 – 2.867)	0.4	1.578 (0.935 – 2.664)	0.087
**Multivariate model**	**sTILs**	High	Ref.		Ref.	
		Low	1.704 (0.7744 – 3.750)	0.18	1.3965 (0.7971 – 2.446)	0.24
	**CD4**	High	Ref		Ref.	
		Low	0.7731 (0.3351 - 1.784)	0.54	1.1955 (0.6484 - 2.204)	0.56
	**CD8**	High	Ref		Ref.	
		Low	0.9804 (0.4344 - 2.213)	0.96	1.6729 (0.9399 - 2.978)	0.08

Supplementary Figure 1. cut-off value calculation CD4 and CD8.

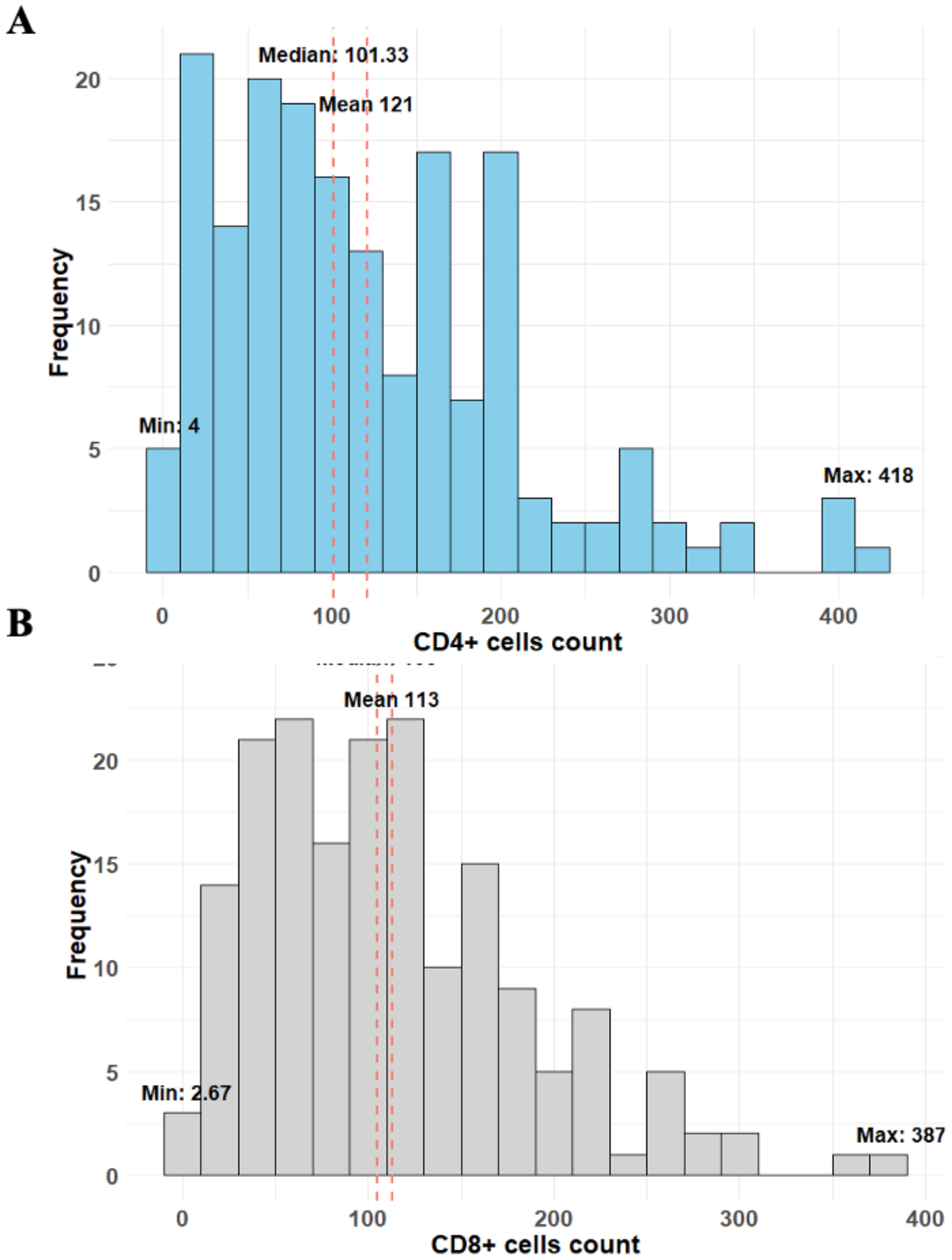

Histogram represent cell counts, mean and median of
(A) CD4, (B) CB8
